# Sleep health among people with severe mental ill health during the COVID-19 pandemic: Results from a linked UK population cohort study

**DOI:** 10.3389/fpsyt.2022.975593

**Published:** 2022-10-10

**Authors:** Paul N. Heron, Lisa M. Henderson, Suzanne Crosland, Simon M. Gilbody, Gordon A. Johnston, Andrew S. Moriarty, Elizabeth Newbronner, Alastair Paterson, Panagiotis Spanakis, Ruth Wadman, Lauren Walker, Emily Peckham

**Affiliations:** ^1^Mental Health and Addictions Research Group, Department of Health Science, University of York, York, United Kingdom; ^2^Department of Psychology, University of York, York, United Kingdom; ^3^Hull York Medical School, York, United Kingdom; ^4^Independent Peer Researcher, Stirling, United Kingdom; ^5^Faculty of Medical and Human Sciences, Manchester University, Manchester, United Kingdom; ^6^Department of Psychology, University of Crete, Rethymmon, Greece; ^7^School of Psychology, Mediterranean College, Athens, Greece

**Keywords:** sleep, severe mental ill health (SMI), bipolar disorder, psychosis, schizophrenia, wellbeing, routine

## Abstract

**Objectives:**

Sleep problems are a transdiagnostic feature of nearly all psychiatric conditions, and a strong risk factor for initial and recurrent episodes. However, people with severe mental ill health (SMI) are often excluded from general population surveys, and as such the extent and associates of poor sleep in this population are less well understood. This study explores sleep health in an SMI sample during the COVID-19 pandemic, using multiple regression to identify risk factors, including daily routine, wellbeing and demographics.

**Methods:**

An existing cohort of people with an SMI diagnosis were sampled. Participants were invited to complete a self-report survey about their health and the impacts of COVID-19 and associated public health measures. Sleep duration, efficiency, and quality were measured using items from the Pittsburgh Sleep Quality Index (PSQI).

**Results:**

Two hundred forty-nine adults (aged 21–84 years) completed the survey. Mean sleep duration and efficiency were similar to general population estimates, at 7 h 19 min and 78%, respectively. However, 43% reported “bad” sleep quality that was associated with being younger in age as well as disturbed routine and declined wellbeing. Indeed, 37% reported a disturbed routine during the pandemic.

**Conclusions:**

High estimates of perceived poor sleep quality in the SMI population align with previous findings. Supporting people with SMI to maintain routine regularity may work to protect sleep quality and wellbeing. Future research should more closely examine sleep health in people with SMI, using accessible and scalable measures of objective and subjective sleep, examining longitudinal trends.

## Introduction

Sleep often serves as an important factor in health, with poor sleep being a persistent and transdiagnostic feature of all psychiatric disorders (1). Sleep disturbances, such as insomnia, circadian rhythm disorders, or behaviors that affect sleep quality, are a risk factor for both initial and recurrent mental health disturbances (2, 3). The importance of understanding the role that sleep plays in mental ill health has heightened during the COVID-19 pandemic, with the government restrictions likely contributing to greater sleep disturbances. Indeed, a metanalysis of sleep problems during COVID-19 and its relation to psychological distress conducted across 39 countries found that sleep problems appeared to be common during the pandemic and were associated with higher degrees of psychological distress (4).

Sleep health is a considerable problem among the people with severe mentally illness (SMI) population, where sleep disorders can have an impact on the onset, course, and treatment of mental ill health (5). This is due, in part, to sleep's regulatory role in various functions of the central nervous system, including mood and cognitive abilities (6). Notably, the rate of sleep disturbance (including insomnia, hypersomnia and delayed sleep phase) in schizophrenia and bipolar disorders has been estimated to be 78% and 69%, respectively, compared to 39% in healthy controls (5). In patients with schizophrenia, studies using polysomnography have shown evidence of insomnia, delayed latency to fall asleep, and reduced total sleep hours and sleep efficiency (7). Circadian rhythm disorders are also common in schizophrenia (8). Disrupted sleep can compound psychotic symptoms and cognitive deficits, and often coincides with acute episodes of psychosis (9). Antipsychotics used in the treatment of schizophrenia can improve sleep problems and quality of life, but are almost without exception sedating which reduces sleep latency and contribute to sleep disturbance (10). NICE guidelines recommend cognitive behavior therapies (CBT) for insomnia over pharmacotherapies where available (11). There is a need for evidence-based interventions for sleep disturbances in schizophrenia, such as CBT which may also help to improve psychotic symptoms (12).

In bipolar disorder, the DSM-5 includes sleep disorders as diagnostic components of both manic and depressive episodes (13). Sleep disturbances are frequent and persistent; insomnia, hypersomnia, and circadian rhythm disorders are common and these disturbances are associated with functional impairment (14). Further, sleep disturbance is a feature of inter-episodic periods, with 70% of euthymic patients with bipolar disorders found to exhibit a clinically significant sleep disturbance, including decreased sleep efficiency, higher anxiety about poor sleep and a tendency to misperceive sleep (15). Actigraphic studies have found that people with bipolar disorder have more night-to-night variability and longer sleep onset latency than healthy comparison groups, as well as increased fragmentation of the sleep/wake cycle (16). There is also an increased prevalence of primary sleep disorders among people with bipolar disorder, including sleep-disturbed breathing (SDB) and obstructive sleep apnoea syndrome (OSAS). The increased incidence of OSAS is at least partly attributable to the effects of psychiatric medications, such as atypical antipsychotics, which are known to contribute to obesity and metabolic syndrome (7). However, relative to schizophrenia, and particularly other psychiatric disorders (e.g., anxiety and depression), there is limited understanding of the role that sleep disturbances play in the initiation, longevity and intervention effectiveness of bipolar disorder, with calls to advance research in this domain (14).

During COVID-19, those with pre-existing mental ill health may have experienced a worsening of symptoms and quality of life (19). Moreover, COVID-19 may pose a greater health risk to the SMI population due to an increased prevalence of medical comorbidities (20) and higher smoking prevalence (21), both of which can negatively impact the prognosis of a Covid infection (22). This increased risk may worsen anxiety symptoms which are commonly associated with sleep disturbances (23). A further key risk factor for worsening sleep during the pandemic is reduced daytime activity and changes in daily routine, owing to the public health restrictions during “lockdown” periods. Previous studies have found that reduced daytime activity contributes to sleep disturbance in schizophrenia (12). Considering these factors, sleep disorders may have increased in prevalence in the SMI population during the COVID-19 pandemic. Given the profound health inequalities already experienced by this population (24), it is important to explore trends in sleep health in this population during the pandemic, not only to ensure that targets for intervention are identified. However, available evidence on the impact of COVID-19 on sleep in the SMI population is limited. To our knowledge, one cross-sectional analysis conducted among people with schizophrenia during COVID-19 found that psychological distress mediated an association between social anxiety and greater sleep disturbances (25). However, these findings come from a single-site study in Taiwan that did not confirm participants' diagnoses and so generalisability is limited. Thus, despite calls for more sleep-related interventions for people with SMI, we need further understanding of the nature and associates of sleep disturbance in this population, especially if we are to increase resilience in the face of future stressogenic situations. Examining sleep in SMI during the pandemic also allows us to capitalize on the natural and rich variation in key risk factors of poor sleep (namely changes in the daily routine and wellbeing).

## Materials and methods

### Design

Data are drawn from the Optimizing Wellbeing During Self-isolation (OWLS) study which is part of the Closing the Gap (CtG) programme of work. CtG is a large transdiagnostic clinical cohort study (*n* = 9,914), the composition of which is described elsewhere (26). A sample of the CtG cohort were invited to take part in OWLS based on gender, age, ethnicity and whether they were recruited *via* primary or secondary care. Those selected to be invited were contacted by telephone or letter and invited to take part in the OWLS study. To be eligible to take part in the OWLS study participants had to be: (i) aged 18 and above, (ii) have documented diagnoses of schizophrenia or delusional/psychotic illness (ICD 10 F20.X & F22.X or DSM equivalent) or bipolar disorder (ICD F31.X or DSM-equivalent). Those who agreed to take part were provided with a range of options: (i) to carry out the survey over the phone with a researcher, (ii) to be sent a link to complete the survey online, or (iii) to be sent a hard copy of the questionnaire in the post to complete and return. The full methods of recruitment to the OWLS study have been previously described (26) and are also outlined in the Supplementary material.

Those who took part in the initial OWLS survey (OWLS 1, T1) were asked if they were willing to complete follow up surveys. We attempted to contact all those who consented to follow up surveys to take part in the follow up survey (OWLS 2, T2). OWLS 2 recruited participants between January 2021 and March 2021.

Ethical approval was granted by the Health Research Authority North West–Liverpool Central Research Ethics Committee (REC reference 20/NW/0276).

### Measures

Where possible, we sought alignment with survey items from the Office of National Statistics (ONS) general population survey to improve comparability. Unless stated, the measures were taken at the OWLS 2 timepoint. Four items from the Pittsburgh Sleep Quality Index (PSQI) were used to measure three sleep sub-domains: subjective sleep quality, sleep duration, and habitual sleep efficiency (17). Participants were asked to report on usual sleep habits within the past month. Variables include: changes to physical and mental health, changes to wellbeing, metabolic equivalent physical activity, time spent sedentary, smoking status, changes to routine, and sociodemographic factors. Table 1 describes the survey items, sources, and interpretation of each variable.

**Table 1:** Variables with respective survey items, responses and interpretation.

**Variable**	**Question**	**Responses**	**Interpretation**
Subjective sleep quality	“In the LAST MONTH, how would you rate your sleep quality overall?” Source: Pittsburgh Sleep Quality Index (PSQI) (17)	Very good; Fairly good	Good sleep quality
		Fairly bad; Very bad	Bad sleep quality
Sleep duration	“In the LAST MONTH, how many hours of actual sleep did you get each night? (hours and minutes) Source: PSQI		Hours and minutes of sleep per night
Habitual sleep efficiency	“During the past month, when have you usually got up in the morning?” and “In the LAST MONTH, at what time have you usually gone to bed at night?” Source: PSQI		= (sleep duration/hours in bed)*100
Decline in physical health	“Compared with 6 MONTHS AGO, how would you rate your physical health in general?”	Worse than before	Deterioration
		Better than before; About the same	No deterioration
Decline in mental health	“Compared with 6 MONTHS AGO, how would you rate your mental health in general?”	Worse than before	Deterioration
		About the same; Better than before	No deterioration
Change in wellbeing	ONS 4-item wellbeing scale, measured at both OWLS 1 & 2 (Supplementary material)	Individual timepoint score range = 0–40	OWLS 2 score minus OWLS 1 score (range−40–40). Higher score = greater wellbeing. A negative score indicates a worsening in wellbeing and a positive score indicates an improvement in wellbeing
Metabolic equivalent (MET) hours of physical activity per day	Six items from International Physical Activity Questionnaire –SF (IPAQ) (18)		MET hours per day
Sedentary hours per day	“In an average week in the LAST MONTH, how much time did you spend per day sitting or lying down, aside of sleeping (e.g. watching TV, sitting in front of computer, etc.?”		Sedentary hours per day
Smoking status	“I smoke…”	More than usual; About the same; Less than usual	Smokes
		I don't do that in general	Does not smoke
Number of cigarettes smoked	“How many cigarettes per day do you usually smoke?” (please choose an option)	10 or less	20 or less
		11 to 20	11 to 20
		21 to 30	21 to 30
		31 or more	31 or more
Deterioration in routine	“In the LAST MONTH, have you been able to maintain a daily routine in your life?”	Less than usual	Deterioration
		About the same; More than usual	No deterioration
Care setting	“Have you needed to use community mental health services?” Source: OWLS 1 survey, regarding since the pandemic restrictions began	Yes	Secondary care
		No	Primary care
Primary mental health diagnosis	Source: participant's medical records. The “Other” diagnosis category contains participants who met the eligibility criteria but whose diagnosis was not specified.		Psychosis-spectrum disorder; Bipolar disorder; Schizoaffective disorder; Other
Index of Multiple Deprivation (IMD)	Source: Postcode obtained at point of inception to CtG study. Measure of deprivation assigned by the Ministry of Housing, Communities and Local Government as a decile (https://imd-by-postcode.opendatacommunities.org/imd/2019)	1 2	1
		3 4	2
		5 6	3
		7 8	4
		9 10	5

### Analysis

The study analysis plan was registered on Open Science Framework (available at https://osf.io/cmgw7). Analyses were undertaken using SPSS v.27. Descriptive statistics were used to describe sleep, health, and sociodemographic characteristics.

To examine associations between the independent variables (physical health deterioration, mental health deterioration, change in wellbeing, MET physical activity, sedentary hours, smoking status, and decline in routine) and sleep duration and habitual sleep efficiency, we used multiple linear regression and we controlled for age, gender, ethnicity, socioeconomic deprivation, care setting, and diagnosis. To examine associations between the aforementioned independent variables and subjective sleep quality (whilst controlling for the same factors), we used a binary logistic regression. Associations of all independent variables with the dependent variable were first examined with a univariable regression analysis. Only variables with *p* < 0.2 in univariate models were included in multivariate models to filter out irrelevant variables (27). All independent variables were inserted all together at once in the multivariable models.

The calculation of the variable MET hours of physical activity per day deviated from the initial analysis plan; upon consultation with physical activity experts then “minutes of walking per day” was not included in the calculation to avoid double counting activity.

## Results

The OWLS 2 survey was completed by 249 participants. 57% (*n* = 142) of participants reported good sleep quality and 43% (*n* = 107) reported bad quality. The mean sleep duration was 7 h, 19 min and the mean sleep efficiency was 78%. Descriptive statistics are presented in Table 2.

**Table 2:** Descriptive statistics for sociodemographic, health and sleep variables.

**Factor**	***N*** **(%)**
**Gender**	
Men	128 (51.4)
Women	116 (46.6)
Transgender	5 (2)
**Age** (mean, range)	51.67, 21–84 years (Median: 52 years, interquartile range: 41–64 years)
**Ethnicity**	
White	210 (87.9)
Non-white	26 (15.2)
**Diagnosis**	
Psychosis-spectrum disorder	120 (48.2)
Bipolar disorder	83 (33.3)
Other SMI	16 (6.4)
Not recorded	30 (12)
**IMD**	
Very high deprivation	60 (24.1)
High deprivation	51 (20.5)
Moderate deprivation	49 (19.7)
Low deprivation	43 (17.3)
Very low deprivation	38 (15.3)
**Mental health care setting**	
Primary care	95 (38.2)
Secondary care	152 (61)
**Physical health**	
Deterioration	95 (38.2)
No deterioration	150 (60.2)
**Mental health**	
Deterioration	85 (34.1)
No deterioration	157 (63.1)
**Wellbeing change** (mean, SD)	−0.48 (7.53)
**Smoking status**	
Smokes	77 (30.9)
Does not smoke	170 (68.3)
**Number of cigarettes per day**	
10 or less	30 (40)
11 to 20	23 (29.9)
21 to 30	13 (16.9)
31 or more	9 (11.7)
**Daily routine**	
Declined routine	91 (36.5)
No declined routine	154 (61.8)
**MET hours of physical activity per day** [hours and minutes (SD)]	4 h, 7 min (5 h, 8 min) Median: 2 h, 46 min, interquartile range: 0 h 28 min – 6 h 12 min
**Sedentary hours per day** [hours and minutes, (SD)]	8 h 38 min (4 h 46 min)
**Sleep quality**	
Very good	56 (22.5)
Fairly good	86 (34.5)
Fairly bad	61 (24.5)
Very bad	46 (18.5)
**Sleep duration** [mean hours and minutes, (SD)]	7 h 19 min (2 h 19 min)
**Sleep efficiency** (%, SD)	78.16 (19.33)

A linear regression on sleep efficiency violated the heterogeneity of variance assumption so we performed a bootstrapped regression analysis. Multivariate models demonstrated that bad sleep quality was associated with being younger in age (adjusted OR = 0.957, 95%CI 0.927–0.988, *p* = 0.008), a decline in routine (adjusted OR = 3.968, 95%CI 1.408–0.11.177, *p* = 0.009), and a worsening in wellbeing (adjusted OR = 0.923, 95%CI 0.857–0.993, *p* = 0.032). Bad sleep quality was reported by 68% of participants in the 18–30 age group (*n* = 28) compared to 39% in the 31–45 age group (*n* = 64), 41% in the 46–65 age group, and 39% in the 66+ age group. Shorter sleep duration was associated with more time spent sedentary (adjusted *B* = −0.124, *p* = 0.015). Poorer sleep efficiency was associated with women (adjusted *B* = −7.918, *p* = 0.025), whereas greater sleep efficiency was associated with having an “other SMI” diagnosis (adjusted *B* = −22.297, *p* = 0.0087). Multivariate models showed that sleep quality, duration, nor efficiency were associated with physical health deterioration, mental health deterioration, smoking, or physical activity. Associations for sleep quality are presented in Table 3, for sleep duration in Table 4, and for sleep efficiency in Table 5.

**Table 3:** Associations between health and socioeconomic variables and sleep quality.

	***N*** **(%)**		**Univariate model**		**Multivariate model**	
	**Good sleep quality**	**Bad sleep quality**	**Odds ratio [Exp (*B*)] (95%CI)**	**P**	**Odds ratio [Exp (*B*)] (95%CI)**	**P**
**Physical health**						
No deterioration	101 (67.3)	49 (32.7)	0.353 (0.207–0.6)	<0.001	0.543 (0.184–1.606)	0.27
Deterioration	40 (42.1)	55 (57.9)	1		1	
**Mental health**						
No deterioration	110 (70.1)	47 (29.9)	0.233 (0.133–0.408)	<0.001	0.373 (0.125–1.117)	0.078
Deterioration	30 (35.3)	55 (64.7)	1		1	
**Wellbeing change**	Mean: 0.58 SD: 7.79	Mean: −1.91 SD: 6.95	0.954 (0.92–0.99)	0.012	0.923 (0.857–0.993)	0.032
**MET physical activity**	Mean: 4.69 SD: 5.13	Mean: 3.39 SD: 5.07	0.946 (0.894–1.001)	0.056	0.987 (0.91–1.071)	0.756
**Time spent sedentary**	Mean: 1.76 SD: 0.43	Mean: 1.46 SD: 0.5	1.117 (1.042–1.198)	0.002	1.089 (0.971–1.221)	0.146
**Smoking status**						
Does not smoke	101 (59.4)	69 (40.6)	1.354 (0.788–2.328)	0.273		
Smokes	40 (51.9)	37 (48.1)	1			
**Maintain routine**						
Decline	34 (37.36)	57 (62.64)	3.592 (2.086–6.186)	<0.001	3.968 (1.408–11.177)	0.009
No decline	105 (68.18)	49 (31.82)	1		1	
**Gender**						
Men	79 (61.72)	49 (38.28)	0.642 (0.386–1.069)	0.088	0.85 (0.3–2.411)	0.76
Women	59 (50.86)	57 (49.14)	1		1	
**Ethnicity**						
Non-white	26 (66.7)	13 (33.3)	1.621 (0.79–3.327)	0.188	1.257 (0.297–5.319)	0.756
White	116 (55.2)	94 (44.8)	1			
**Age**	Mean: 53.47 SD: 14.22	Mean: 49.3 SD: 15.85	0.981 (0.965–0.998)	0.031	0.957 (0.927–0.988)	0.008
**IMD**						
Very high	30 (50)	30 (50)	2.167 (0.925–5.074)	0.075	2.331 (0.487–11.164)	0.289
High	28 (54.9)	23 (45.1)	1.78 (0.739–4.285)	0.198	0.955 (0.198–4.599)	0.954
Medium	30 (61.2)	19 (38.8)	1.372 (0.562–3.353)	0.488	0.319 (0.062–1.632)	0.17
Low	25 (58.1)	18 (41.9)	1.56 (0.626–3.89)	0.34	1.033 (0.219–4.883)	0.967
Very low	26 (68.4)	12 (31.6)	1		1	
**Care setting**						
Secondary care	91 (59.9)	61 (40.1)	0.714 (0.426–1.197)	0.201	0.708 (0.261–1.922)	0.498
Primary care	49 (51.6)	46 (48.4)	1		1	
**Diagnosis**						
Not recorded	20 (66.7)	10 (33.3)	0.833 (0.358–1.939)	0.672	0.208 (0.031–1.382)	0.104
Other SMI	5 (37.5)	10 (62.5)	2.778 (0.946–8.159)	0.063	3 (0.307–29.298)	0.345
Bipolar	41 (49.4)	42 (50.6)	1.707 (0.968–3.011)	0.065	1.404 (0.476–4.139)	0.538
Psychosis	75 (62.5)	45 (37.5)	1	0.075	1	0.199

**Table 4:** Associations between health and socioeconomic variables and sleep duration.

	**Univariate model**		**Multivariate model**	
	***B*** **(standard error)**	**P**	***B*** **(standard error)**	**P**
**Physical health**				
No deterioration	−0.805 (0.3)	0.008	0.013 (0.485)	0.979
Deterioration	1		1	
**Mental health**				
No deterioration	−0.986 (0.309)	0.002	0.154 (0.511)	0.763
Deterioration	1		1	
**Wellbeing change**	0.011 (0.02)	0.575		
**MET physical activity**	0.068 (0.029)	0.019	0.026 (0.038)	0.495
**Time spent sedentary**	−0.133 (0.038)	<0.001	−0.124 (0.05)	0.015
**Smoking status**				
Smokes	0.056 (0.321)	0.862		
Does not smoke	1			
**Maintain routine**				
Decline	0.835 (0.304)	0.006	0.446 (0.469)	0.343
No decline	1		1	
**Gender**				
Men	−0.398 (0.297)	0.182	−0.218 (0.438)	0.62
Women	1		1	
**Ethnicity**				
Non-white	0.679 (0.406)	0.096	−0.059 (0.585)	0.92
White	1		1	
**Age**	0.001 (0.01)	0.881	0.003 (0.014)	0.84
**IMDD**				
Very high	−0.445 (0.482)	0.356	−0.805 (0.711)	0.26
High	−0.238 (0.501)	0.635	−0.36 (0.716)	0.616
Medium	−0.088 (0.505)	0.862	−0.266 (0.679)	0.696
Low	0.066 (0.521)	0.899	−0.058 (0.697)	0.934
Very low	1		1	
**Care setting**				
Secondary care	−0.834 (0.304)	0.007	−0.906 (0.447)	0.045
Primary care	1		1	
**Diagnosis**				
Not recorded	−0.779 (0.473)	0.101	−0.008 (0.669)	0.991
Other SMI	−1.661 (0.607)	0.007	−1.74 (0.973)	0.076
Bipolar	−0.545 (0.33)	0.1	−0.429 (0.478)	0.371
Psychosis	1		1	

**Table 5:** Associations between health and socioeconomic variables and habitual sleep efficiency.

	**Univariate model**		**Multivariate model**	
	***B*** **(standard error)**	**P**	***B*** **(standard error)**	**P**
**Physical health**				
No deterioration	−7.517 (2.555)	0.004	−0.49 (3.871)	0.899
Deterioration	1		1	
**Mental health**				
Deterioration	−9.085 (2.667)	<0.001	−1.913 (4.016)	0.635
No deterioration	1		1	
**Wellbeing change**	0.122 (0.172)	0.478		
**MET physical activity**	0.564 (0.246)	0.023	0.226 (0.305)	0.46
**Time spent sedentary**	−0.462 (0.326)	0.159	−0.15 (0.397)	0.706
**Smoking status**				
Smokes	−1.589 (2.78)	0.568		
Does not smoke	1			
**Maintain routine**				
Decline	6.476 (2.651)	0.015	1.636 (3.775)	0.666
No decline	1		1	
**Gender**				
Men	−4.977 (2.553)	0.052	−7.918 (3.495)	0.025
Women	1		1	
**Ethnicity**				
Non-white	6.235 (3.491)	0.075	−2.525 (4.593)	0.584
White	1		1	
**Age**	−0.068 (0.085)	0.425	−0.088 (0.113)	0.437
**IMD**				
Very high	−0.869 (4.07)	0.831	−8.905 (5.606)	0.115
High	−4.139 (4.278)	0.334	−9.616 (5.676)	0.093
Medium	4.402 (4.3)	0.307	1.264 (5.435)	0.817
Low	−0.955 (4.397)	0.828	−2.522 (5.456)	0.645
Very low	1		1	
**Care setting**				
Secondary care	−4.722 (2.636)	0.075	−5.379 (3.568)	0.134
Primary care	1		1	
**Diagnosis**				
Not recorded	−5.931	0.139	−1.078 (5.252)	0.838
Other SMI	−18.588	<0.001	−22.297 (8.173)	0.007
Bipolar	−4.693	0.094	−4.103 (3.779)	0.28
Psychosis	1		1	

## Discussion

The present study represents a novel analysis of sleep health in UK adults with SMI, addressing the extent to which subjective sleep parameters (namely, quantity, quality and efficiency) are associated with key stressors that were impacted by the pandemic.

There were mixed findings regarding sleep health in the present sample. The mean self-reported daily sleep duration was ~7 h and 19 min per day (with mean efficiency of 78%). These estimates are close to the mean 7.2 h of sleep reported per day for general population samples (28) and are similar to what other SMI studies report. Aschbrenner et al. (29) found that 69% of young adults with SMI reported 7 h or more of sleep per night, and 64% reported a sleep efficiency >75% (29); Keskin et al. (30) found that mean sleep duration in people with euthymic bipolar disorder was >8 h).

Despite these healthy levels of sleep duration and efficiency, two in five (43%) participants reported experiencing bad or very bad sleep quality. Compared to a metanalysis of general population studies conducted during COVID-19 where the pooled prevalence of sleep problems were 18% (4), perceived sleep problems were considerably more prevalent in this SMI sample. Poor sleep quality has previously been reported among other SMI samples; Aschbrenner et al. (29) found that 36% reported poor sleep quality, and Keskin et al. (30) found that 57% reported poor sleep quality. Although these studies offer valuable opportunities to which the present findings can be compared, the method of defining poor sleep quality varies by study, so these comparisons should be viewed with caution. For example, Keskin et al. (30) defined poor sleep quality according to the full 18 item PSQI score, whereas the present study defined it according to the sleep-quality sub-domain of the PSQI only.

One in three (37%) participants reported a decline in daily routine since an earlier pandemic stage and this was strongly associated with poor sleep quality and declined wellbeing. The pandemic has impacted many people's daily routine, for example by reducing working hours and the freedom to leave one's home. Analysis of big data indicates that the circadian rhythm of society has changed during COVID-19 as a result of changes to daily routine, with greater technology use later in the day, and changes to internet traffic that indicate later bedtimes and less overall sleep (31). Maintaining regularity in lifestyle may help to regulate the circadian rhythm and this helps to explain why people with insomnia report less regularity in their daily routines compared to those without insomnia (32). There is also evidence that sleep timing and irregularity is linked to greater daytime sleepiness and poorer sleep quality (33). Chiming with the present data, in a study of 91,105 participants in the UK Biobank population, disruption to rest-activity cycles was associated with increased risk of depression, bipolar disorder, neuroticism, low wellbeing and lower health satisfaction, even when controlling for a number of demographic and environmental variables (34). Thus, it is possible that the pandemic has negatively affected people's ability to maintain a daily routine which may have worsened sleep quality and wellbeing. However, there were no comparative measures of sleep or routine regularity outside of the pandemic time period. Additionally, it may be that a decline in wellbeing and in sleep quality led to a declined ability to maintain a daily routine, but causality cannot be determined in the present findings. The concept of daily routine used in this study is also general and does not differentiate between work, social or other types of routines. Lower social regularity was measured in people with bipolar disorder prior to the pandemic (35, 36), and was associated with greater depressive symptoms among patients with mood disorders during the pandemic (37); given these associations, it would be valuable to explore associations between sleep health and the regularity of different types of routine. Notwithstanding this, the present findings confirm that maintaining a regular routine may be an important target for intervention both to improve sleep and wellbeing in the SMI population.

In the present sample, a number of demographic variables were associated with poor sleep. For instance, poor sleep quality was associated with being younger in age; 68% of participants aged 18–30 years reported bad sleep compared to 39%−41% across all other age groups. General population studies also report that younger people experience poorer sleep quality (38). Poor sleep efficiency was also associated with women. This supports previous findings that women with schizophrenia are more likely than women without schizophrenia to report sleep reduced sleep efficiency (39). It also resonates with findings of greater female susceptibility to sleep difficulties in other disorders. For example, females with autism are at greater risk of poor sleep efficiency than males (40), with claims that an increased incidence of internalizing difficulties in females with autism may put them at greater risk of sleep difficulties (or vice versa) (41).

Despite the commonly-reported association between sleep and mental ill health (23), the present analysis found no significant associations between sleep factors and a self-reported deterioration in mental health since an earlier phase of the pandemic. However, poor sleep quality (but not sleep duration/efficiency) was associated with declines in the 4-item wellbeing measure during the pandemic. Similarly, Gould et al. (42) found that subjective quality of sleep (also measured *via* the PSQI) was a stronger predictor of depression and anxiety in an aging adult population than objective measures of sleep architecture (including total sleep time). One possibility here is that perceived sleep quality captures subtle difficulties with sleep (e.g., sleep onset problems, bedtime anxiety) better than quantity-based measures, or alternatively that perception of poor sleep is a further symptom of psychiatric disorders that is independent of objective sleep. Further research is needed to address these possibilities.

### Strengths and limitations

The recruitment method resulted in a sample highly representative of its population, and explores both psychosis spectrum and bipolar disorders. The survey examined a range of health domains which allowed a variety of factor associations to be explored. The present study therefore provides a useful broad exploration of current sleep trends in SMI populations and offers recommendations for a more in-depth analysis of this subject. However, there are also important limitations to consider. For instance, psychiatric symptoms are closely-related to sleep and routine regularity and could explain variance in sleep outcomes, but symptoms were not measured in this study. Additionally, it was not possible to compare sleep health prior to the pandemic to sleep health during the pandemic as no pre-pandemic sleep measures had been collected from our sample. However, that wellbeing and daily routine were measured as change since an earlier phase in the pandemic improved contextualization of the findings to the pandemic period. Utilizing the PSQI was advantageous because its wide usage allowed comparison of present with previous findings. However, the PSQI does not capture daytime sleepiness or napping which are commonly reported among SMI populations (43). Further, future studies are needed that utilize scalable measures of both subjective and objective measures of sleep, capturing both trait and state features, to ascertain a richer picture of sleep health in this population.

## Conclusion

Relatively little is known about sleep health among SMI populations, compared to other psychiatric disorders. The present study shows that poor sleep quality is a common experience for people with SMI and it is associated with declines in routine regularity and wellbeing. One third of participants reported a decline in routine regularity during the pandemic and this may have impacted on sleep quality. Future research should explore the impact of sleep trends by collecting longitudinal sleep data in SMI samples. Sleep health is complex, particularly in this population, and studies would benefit from qualitative exploration or more comprehensive evaluations of sleep health. Supporting people with SMI to maintain regularity in daily routine may reap important benefits to sleep quality and to wellbeing. Indeed, poor sleep has historically been neglected as a specific treatment target in mental health programmes (44). However, targeting sleep difficulties alongside mainstream interventions should be examined as an effective and scalable model for improving outcomes (45).

## Data availability statement

The datasets presented in this study can be found in online repositories. The names of the repository/repositories and accession number(s) can be found at: http://doi.org/10.5255/UKDA-SN-855270.

## Ethics statement

The studies involving human participants were reviewed and approved by Health Research Authority North West–Liverpool Central Research Ethics Committee (REC reference 20/NW/0276). Written informed consent for participation was not required for this study in accordance with the national legislation and the institutional requirements.

## Author contributions

PH, SC, EN, PS, LW, and EP contributed to data collection. PH, PS, and EP contributed to the data analysis. All authors contributed to the development and write-up of the study.

## Funding

EP, PS, PH, GJ, EN, and SG Medical Research Council (grant reference MR/V028529) https://mrc.ukri.org/funding/. SG Economic and Social Research Council (ES/S004459/1) https://www.ukri.org. SG, RW, EN, and PS National Institute for Health Research (NIHR200166) https://www.nihr.ac.uk.

## Conflict of interest

The authors declare that the research was conducted in the absence of any commercial or financial relationships that could be construed as a potential conflict of interest.

## Publisher's note

All claims expressed in this article are solely those of the authors and do not necessarily represent those of their affiliated organizations, or those of the publisher, the editors and the reviewers. Any product that may be evaluated in this article, or claim that may be made by its manufacturer, is not guaranteed or endorsed by the publisher.

## References

[1] Harvey AG Murray G Chandler RA Soehner A. Sleep disturbance as transdiagnostic: Consideration of neurobiological mechanisms. Clin Psychol Rev. (2011) 31:225–35. 10.1016/j.cpr.2010.04.003 20471738 PMC2954256

[2] Freeman D Sheaves B Goodwin GM Yu L-M Nickless A Harrison PJ. The effects of improving sleep on mental health (OASIS): a randomised controlled trial with mediation analysis. Lancet Psychiatry. (2017) 4:749–58. 10.1016/S2215-0366(17)30328-0 28888927 PMC5614772

[3] Harvey AG. Insomnia: symptom or diagnosis? Clin Psychol Rev. (2001) 21:1037–59. 10.1016/S0272-7358(00)00083-0 11584515

[4] Alimoradi Z Broström A Tsang HWH Griffiths MD Haghayegh S Ohayon MM. Sleep problems during COVID-19 pandemic and its' association to psychological distress: a systematic review and meta-analysis. EclinicalMedicine. (2021) 36:100916. 10.1016/j.eclinm.2021.100916 34131640 PMC8192091

[5] Laskemoen JF Simonsen C Büchmann C Barrett EA Bjella T Lagerberg TV. Sleep disturbances in schizophrenia spectrum and bipolar disorders – a transdiagnostic perspective. Compr Psychiatry. (2019) 91:6–12. 10.1016/j.comppsych.2019.02.006 30856497

[6] Gica S Selvi Y. Sleep interventions in the treatment of schizophrenia and bipolar disorder. Noro psikiyatri arsivi. (2021) 58:S53–60. 10.29399/npa.27467 34658636 PMC8498809

[7] Benca RM Obermeyer WH Thisted RA Gillin JC. Sleep and psychiatric disorders. A meta-analysis. Arch Gen Psychiatry. (1992) 49:651–68. discussion 669–670. 10.1001/archpsyc.1992.01820080059010 1386215

[8] Wulff K Joyce E. Circadian rhythms and cognition in schizophrenia. Br J Psychiatry. (2011) 198:250–2. 10.1192/bjp.bp.110.085068 21972273

[9] Sprecher KE Ferrarelli F Benca RM. Sleep and plasticity in schizophrenia. Curr Top Behav Neurosci. (2015) 25:433–58. 10.1007/7854_2014_366 25608723 PMC5117633

[10] Krystal AD Goforth HW Roth T. Effects of antipsychotic medications on sleep in schizophrenia. Int Clin Psychopharmacol. (2008) 23:150–60. 10.1097/YIC.0b013e3282f39703 18408529

[11] National Institute for Health and Care Excellence (NICE). (2022). Scenario: Managing long-term insomnia (more than 3 months duration). Available online at: https://cks.nice.org.uk/topics/insomnia/management/managing-long-term-insomnia-greater-3-months/ (accessed 16 June 2022).

[12] Waite F Myers E Harvey AG Espie CA Startup H Sheaves B. Treating Sleep problems in patients with schizophrenia. Behav Cogn Psychother. (2016) 44:273–87. 10.1017/S1352465815000430 26751571 PMC4855992

[13] APA. (2013). American Psychiatric Association: Diagnostic and Statistical Manual of Mental Disorders (DSM-5), 5th ed. Washington, DC: American Psychiatric Publishing. Available online at: https://www.psychiatry.org/psychiatrists/(accessed 16 June 2022).

[14] Kaplan KA. Sleep and sleep treatments in bipolar disorder. Curr Opin Psychol. (2020) 34:117–22. 10.1016/j.copsyc.2020.02.001 32203912

[15] Harvey AG Schmidt DA Scarnà A Semler CN Goodwin GM. Sleep-related functioning in euthymic patients with bipolar disorder, patients with insomnia, and subjects without sleep problems. Am J Psychiatry. (2005) 162:50–7. 10.1176/appi.ajp.162.1.50 15625201

[16] Millar AL Espie CA Scott J. The sleep of remitted bipolar outpatients: a controlled naturalistic study using actigraphy. J Affect Disord. (2004) 80:145–53. 10.1016/S0165-0327(03)00055-7 15207927

[17] Buysse DJ Reynolds CF 3rd Monk TH Berman SR Kupfer DJ. The Pittsburgh Sleep Quality Index: a new instrument for psychiatric practice and research. Psychiatry Res. (1989) 28:193–213. 10.1016/0165-1781(89)90047-4 2748771

[18] Craig CL Marshall AL Sjöström M Bauman AE Booth ML Ainsworth BE. International physical activity questionnaire: 12-country reliability and validity. Med Sci Sports Exerc. (2003) 35:1381–95. 10.1249/01.MSS.0000078924.61453.FB 12900694

[19] Sheridan Rains L Johnson S Barnett P Steare T Needle JJ Carr S. Early impacts of the COVID-19 pandemic on mental health care and on people with mental health conditions: framework synthesis of international experiences and responses. Soc Psychiatry Psychiatr Epidemiol. (2021) 56:13–24. 10.1007/s00127-020-01924-7 32804258 PMC7429938

[20] Bahorik AL Satre DD Kline-Simon AH Weisner CM Campbell CI. Serious mental illness and medical comorbidities: findings from an integrated health care system. J Psychosom Res. (2017) 100:35–45. 10.1016/j.jpsychores.2017.07.004 28789791 PMC5576509

[21] Szatkowski L McNeill A. Diverging trends in smoking behaviors according to mental health status. Nicotine Tob Res. (2015) 17:356–60. 10.1093/ntr/ntu173 25180078 PMC5479511

[22] Espejo-Paeres C Núñez-Gil IJ Estrada V Fernández-Pérez C Uribe-Heredia G Cabré-Verdiell C. Impact of smoking on COVID-19 outcomes: a HOPE Registry subanalysis. BMJ Nutr Prev Health. (2021) 4:285–92. 10.1136/bmjnph-2021-000269 34308137 PMC8214987

[23] Alvaro PK Roberts RM Harris JK. A systematic review assessing bidirectionality between sleep disturbances, anxiety, and depression. Sleep. (2013) 36:1059–68. 10.5665/sleep.2810 23814343 PMC3669059

[24] Hayes JF Marston L Walters K King MB Osborn DPJ. Mortality gap for people with bipolar disorder and schizophrenia: UK-based cohort study 2000-2014. Br J Psychiatry. (2017) 211:175–81. 10.1192/bjp.bp.117.202606 28684403 PMC5579328

[25] Li D-J Chou L-S Chou FH-C Hsu S-T Hsieh K-Y Wu H-C. COVID-related psychological distress fully mediates the association from social impact to sleep disturbance among patients with chronic schizophrenia. Sci Rep. (2021) 11:16524–16524. 10.1038/s41598-021-96022-2 34400716 PMC8368012

[26] Peckham E Allgar V Crosland S Heron P Johnston G Newbronner E. Health risk behaviours among people with severe mental ill health during the COVID-19 pandemic: analysis of linked cohort data. PloS ONE. (2021) 16:e0258349. 10.1371/journal.pone.0258349 34648548 PMC8516268

[27] Vittinghoff E Glidden DV Shiboski SC McCulloch CE. Regression Methods in Biostatistics. New York, NY: Springer-Verlag (2012).

[28] Dashti HS Jones SE Wood AR Lane JM van Hees VT Wang H. Genome-wide association study identifies genetic loci for self-reported habitual sleep duration supported by accelerometer-derived estimates. Nat Commun. (2019) 10:1100. 10.1038/s41467-019-08917-4 30846698 PMC6405943

[29] Aschbrenner KA Naslund JA Salwen-Deremer JK Browne J Bartels SJ Wolfe RS. Sleep quality and its relationship to mental health, physical health and health behaviours among young adults with serious mental illness enrolled in a lifestyle intervention trial. Early Interv Psychiatry. (2022) 16:106–10. 10.1111/eip.13129 33594828 PMC10047807

[30] Keskin N Tamam L Ozpoyraz N. Assessment of sleep quality in bipolar euthymic patients. Compr Psychiatry. (2018) 80:116–25. 10.1016/j.comppsych.2017.09.012 29091777

[31] Paterson A. A Month of Sundays? What technology Can Tell us About Sleep on Lockdown. (2020). 10.47795/WKFS4313

[32] Moss TG Carney CE Haynes P Harris AL. Is daily routine important for sleep? An investigation of social rhythms in a clinical insomnia population. Chronobiol Int. (2015) 32:92–102. 10.3109/07420528.2014.956361 25187987

[33] Irish LA Kline CE Gunn HE Buysse DJ Hall MH. The role of sleep hygiene in promoting public health: a review of empirical evidence. Sleep Med Rev. (2015) 22:23–36. 10.1016/j.smrv.2014.10.001 25454674 PMC4400203

[34] Lyall LM Wyse CA Graham N Ferguson A Lyall DM Cullen B. Association of disrupted circadian rhythmicity with mood disorders, subjective wellbeing, and cognitive function: a cross-sectional study of 91 105 participants from the UK Biobank. Lancet Psychiatry. (2018) 5:507–14. 10.1016/S2215-0366(18)30139-1 29776774

[35] Ashman SB Monk TH Kupfer DJ Clark CH Myers FS Frank E. Relationship between social rhythms and mood in patients with rapid cycling bipolar disorder. Psychiatry Res. (1999) 86:1–8. 10.1016/S0165-1781(99)00019-0 10359478

[36] Shen GH Alloy LB Abramson LY Sylvia LG. Social rhythm regularity and the onset of affective episodes in bipolar spectrum individuals. Bipolar Disord. (2008) 10:520–9. 10.1111/j.1399-5618.2008.00583.x 18452448 PMC4090015

[37] Kahawage P Bullock B Meyer D Gottlieb J Crowe M Swartz HA. Social rhythm disruption is associated with greater depressive symptoms in people with mood disorders: findings from a multinational online survey during COVID-19. Can J Psychiatry. (2022). 10.1177/07067437221097905. [Epub ahead of print]. 35535550 PMC9096005

[38] Gadie A Shafto M Leng Y Kievit RA Cam CAN. How are age-related differences in sleep quality associated with health outcomes? An epidemiological investigation in a UK cohort of 2406 adults. BMJ Open. (2017) 7:e014920–e014920. 10.1136/bmjopen-2016-014920 28760786 PMC5642766

[39] Chen MH Korenic SA Wickwire EM Wijtenburg SA Hong LE Rowland LM. Sex differences in subjective sleep quality patterns in schizophrenia. Behav Sleep Med. (2020) 18:668–79. 10.1080/15402002.2019.1660168 31462084 PMC7047560

[40] Henderson LM St Clair M Knowland V van Rijn E Walker S Gaskell MG. Stronger associations between sleep and mental health in adults with autism: a UK Biobank study. J Autism Dev Disord. (2021). 10.1007/s10803-021-05382-1 34860312 PMC10066094

[41] Jovevska S Richdale AL Lawson LP Uljarevj M Arnold SRC Trollor JN. Sleep quality in autism from adolescence to old age. Autism Adulthood. (2020). 10.1089/aut.2019.0034 36601570 PMC8992849

[42] Gould CE Karna R Jordan J Kawai M Hirst R Hantke N. Subjective but not objective sleep is associated with subsyndromal anxiety and depression in community-dwelling older adults. Am J Geriatr Psychiatry. (2018) 26:806–11. 10.1016/j.jagp.2018.03.010 29709510 PMC6008208

[43] Sharma P Dikshit R Shah N Karia S De Sousa A. Excessive daytime sleepiness in schizophrenia: A naturalistic clinical study. J Clin Diagn Res. (2016) 10:VC06–VC08. 10.7860/JCDR/2016/21272.8627 27891431 PMC5121769

[44] Freeman D Sheaves B Waite F Harvey AG Harrison PJ. Sleep disturbance and psychiatric disorders. Lancet Psychiatry. (2020) 7:628–37. 10.1016/S2215-0366(20)30136-X 32563308

[45] Stott R Pimm J Emsley R Miller CB Espie CA. Does adjunctive digital CBT for insomnia improve clinical outcomes in an improving access to psychological therapies service? Behav Res Ther. (2021) 144:103922. 10.1016/j.brat.2021.103922 34246110

